# Nutritional Improvement of Sugar-Snap Cookies Supplemented with *Ganoderma sessile* and *Pleurotus ostreatus* Solid-State Fermentation Flours

**DOI:** 10.3390/foods15030510

**Published:** 2026-02-02

**Authors:** Antonella Mazzola, Pablo Ribotta, Francisco Kuhar, Fernanda Quiroga, Alina Greslebin

**Affiliations:** 1 Laboratory of Bioprospecting and Applied Research in Plants and Fungi (LaBIAPH), National University of Patagonia SJB, Route 259 Km 16.4, Esquel 9200, Argentina; antomazzola@outlook.com; 2 Institute for Research, Development and Food Services (ISIDSA), National University of Córdoba, Juan Filloy s/n-Ciudad Universitaria, Córdoba 5000, Argentina; pdribotta@unc.edu.ar; 3 Institute of Food Science and Technology of Córdoba (CONICET-UNC), National University of Córdoba, Juan Filloy s/n-Ciudad Universitaria, Córdoba 5000, Argentina; fquiroga@agro.unc.edu.ar; 4 Multidisciplinary Plant Biology Research Institute (IMBIV, CONICET-UNC), National University of Córdoba, Av. Vélez Sarsfield 1666, Córdoba 5000, Argentina; fkuhar@gmail.com

**Keywords:** antioxidant, functional foods, polyphenols, protein, triterpene

## Abstract

Wheat and rice are major sources of human nutrition worldwide. Solid-state fermentation (SSF) with lignocellulolytic mushrooms can enhance their nutritional value and increase their functional properties. However, this technology is not yet widely applied. In this work, whole wheat and brown rice hydrated to 60% were used as substrates for the edible mushroom *Pleurotus ostreatus* and the medicinal *Ganoderma sessile*, which were incubated for 14 days at 25 °C in the dark. The fermented substrate biomass was incorporated into standard sugar-snap cookie recipes, substituting 20% of the wheat flour. We evaluated the technological and nutritional properties of alternative fermented flours and cookies. Both the fermented flours and cookies exhibited increased soluble and total protein content, antioxidant power, and phenol content, indicating overall functional improvement. Fermented *G. sessile* flour also showed increased triterpenoid content. The physical quality of cookies remained within expected ranges, demonstrating the feasibility of the application. These results highlight the potential of SSF as a method for nutritional and functional enrichment of grains and extend the health benefits of mushrooms to populations relying on low-cost, grain-derived carbohydrates. Further studies on digestibility and in vivo activity of metabolites are needed to confirm the potential health benefits of fermented flours.

## 1. Introduction

Fermentation is a biological process that transforms substrates into value-added products through the metabolic activity of microorganisms. Among various techniques, solid-state fermentation (SSF) by filamentous fungi has proven particularly effective in nutritionally and functionally enriching substrates. SSF involves cultivating microorganisms on solid substrates without free water [1]. This process enhances the nutritional composition by breaking down complex macromolecules, such as fats, carbohydrates, and proteins, into smaller, more bioavailable compounds [2,3]. Additionally, SSF improves the amino acid profile by increasing the bioavailability of essential amino acids and releasing bioactive substances, such as phenolic compounds and antioxidants [4,5]. The resulting “fermented flours”, obtained by drying and milling cereals fermented with edible and medicinal mushrooms, add nutritional value to a wide range of products [6,7].

Fungi are well known for their health-beneficial properties due to their antioxidant, immunoregulatory, anti-hypertensive, anti-inflammatory, hypocholesterolemic, anti-diabetic, and prebiotic activities [8,9]. Incorporating these fungal species into the production of fermented flours and subsequently using them in bakery products presents an opportunity to create functional foods that satisfy taste and provide health benefits. This approach enables the incorporation of the whole mycelium into food matrices, which has been associated with the presence of a wide range of metabolites and bioactive compounds such as polyphenols and terpenes, among others [10,11,12].

Beyond their nutritional benefits, flours also possess essential technological properties that are critical for functionality in bakery applications. Key characteristics such as particle size, viscosity, water absorption, and moisture content determine the final product’s texture, hydration capacity, and overall quality [13]. For instance, flour’s particle size distribution directly influences the dough’s viscoelastic properties. Also, larger particles tend to absorb water more slowly than finer particles, requiring a longer time for adequate mixing and homogenization [14].

Cookies are a widely consumed bakery product globally, appreciated for their low cost, convenience as a ready-to-eat food, variety of flavors, and extended shelf life. They are produced from short dough with high sucrose and fat content and low water content. Substituting white wheat flour with these fungal fermented alternatives can significantly impact the properties of dough and cookies. For instance, these substitutions can affect dough water absorption, leading to changes in consistency and its tendency to stick to equipment. Additionally, it can alter dough spreading, leading to thicker, denser cookies with a less smooth texture and uneven baking.

During cookie preparation, gluten development is not desired [15]. The use of white wheat flour, characterized by weak gluten strength, along with proper ingredients, allows for a tender texture and proper structural development in baked goods such as cookies, avoiding the adverse effects of high protein content on cookie diameter and texture [16]. However, compared with whole-grain flour, white flour has lower levels of essential nutrients such as fiber, lysine, B-group vitamins, and primary and trace elements [17]. Although refined flours are often enriched with iron, thiamin, riboflavin, niacin, and folic acid before being used in food products, this enrichment only replaces some of the nutrients lost during the refining process. One strategy to address this nutrient loss is to substitute refined flours with sprouted, fermented, or whole flours, which are rich in fiber and specific nutrients. Partial or complete replacement of the base flour with another flour substitute (e.g., mushroom powders) [11], pulse flour [18], or fruit pulp [19] can improve the nutritional composition of blended flour. Still, it may also alter the technological quality of flours and final products. Key quality indicators include oven spreading, final diameter, width-to-height ratio, “cookie top grain” (the grain on the cookie’s surface), and firmness [20]. Alternative flours often have a darker color, which can affect the final appearance of cookies. Furthermore, cookies may become drier or retain moisture differently, depending on the properties of new ingredients used [21,22,23]. Good cookie quality is generally associated with a large diameter, a tender yet snapping final product, and a uniform surface cracking pattern [24].

An evident knowledge gap exists between the biological activities abundantly reported in the literature for these mushroom species and the application in final products and culinary preparations, especially regarding biomass resulting from SSF methodologies, especially in the case of SSF using *Ganoderma* species, which has not been previously addressed from a nutritional and functional point of view. Integrating nutritionally enriched flours, obtained by fermenting grains with *Pleurotus ostreatus* and *Ganoderma sessile*, presents a unique opportunity to enhance the nutritional and bioactive content of cookies. Therefore, this study proposes the hypothesis that incorporating fermented flours from *Ganoderma* and *Pleurotus* species can improve the nutritional profile and help cookies to meet the demands of modern consumers seeking healthier options. The objective of this study was to evaluate the technological and dietary properties of fermented flours obtained through solid-state fermentation of whole wheat and brown rice substrates using strains of *Ganoderma sessile* and *Pleurotus ostreatus* and to analyze the impact of these flours on cookie formulation.

## 2. Materials and Methods

### 2.1. Grains and Flours

Long-grain whole-grain unpolished rice with the bran layer intact (brown rice) (*Oryza sativa*) produced in the province of Entre Ríos (Villaguay, Entre Ríos, Argentine) and hard white whole wheat (*Triticum aestivum*) produced in the province of Chubut (Granja de la Pradera, Las Golondrinas, Chubut, Argentine) were purchased in bulk from the listed producers.

All-purpose commercial wheat flour (Gastaldi™, Córdoba, Argentine) was used (protein 10.4%, moisture 12.0%, ash 0.62% (dry basis), and gluten 23%).

### 2.2. Strains

The *G. sessile* strain E47 (University of Guelph, Canada) and the *P. ostreatus* strain A02 (CERZOS, Bahía Blanca, Argentine), both available from the LaBIAPH strain collection, were used. Colonies were cultured and kept on malt extract agar (MEA) medium. One mycelium agar plug (0.5 cm in diameter) was inoculated into each wet Petri dish (90 mm in diameter) with MEA medium and incubated at 25 °C in the dark for 7 days for *G. sessile* and 10 days for *P. ostreatus*.

### 2.3. Spawn

The spawn was prepared as described in Mazzola et al. [12], using a substrate of cooked brown rice with 60% (*w*/*w*) humidity. Brown rice was boiled in excess of water for 15 min. After being left to drain for 10 min, the final water content of the substrate was determined gravimetrically. Five subsamples of cooked brown rice were dried to constant weight; water content was calculated as the difference between the wet and dry weights. Bags (25 × 30 cm) containing 200 g of these final substrates were sealed with 3.5 cm diameter cotton plugs to allow for gas exchange and were immediately sterilized in an autoclave (121 °C, 122 kPa) for 0:45 h. After cooling, the bags were inoculated in one completely colonized Petri dish (i.e., a mycelial mat of 4.5 cm^2^), prepared as indicated above in Section 2.2, and incubated at 25 °C in the dark for two weeks.

### 2.4. Substrate Preparation

The brown rice and whole wheat grains were hydrated by boiling for 15 and 20 min, respectively, leaving behind 60% (*w*/*w*) and 50% (*w*/*w*) of their final water content, respectively. Final water content was determined as described in Section 2.3. Hydrated substrates were then loosely placed in disposable aluminum trays (13 × 22 cm), each containing 500 g of substrate, resulting in a final substrate bed thickness of 2–2.5 cm. Trays were covered with aluminum foil lids and sterilized in an autoclave (121 °C, 122 kPa) for 1:30 h. No filter to gas exchange was included because the lids allow for gas exchange in the same way as Petri dishes do. Inoculation was performed as described in Mazzola et al. [12] under a horizontal laminar flow hood (HB1500 Biotraza, Buenos Aires, Argentina). Each tray received 50 g (wet basis) of ground spawn suspended in sterile water (1:2 spawn/water ratio). Inoculum was evenly poured over the substrate surface. Six trays of each substrate × species were prepared. Six sterilized, non-inoculated trays of each substrate were kept as controls of the process (S BR and S WW). Incubation was performed at 25 °C in the dark for two weeks. After incubation, all experimental units (hydrated biomass) were dried at 60 °C until reaching a constant mass and then milled in a laboratory mill. The particle size obtained for all fermented flours was D 3,4 < 300 µm, and D10, D50, and D90 < D3,4 (HORIBA laser diffraction analyzer, LA 960, Irvine, CA, USA). Flours were stored in airtight plastic bags in the dark and processed within 6 months of the end of the study. Finally, two composite samples of each fermented flour (substrate × species) and control were prepared by mixing the flour of three trays (replicates). The drying temperature was set at 60 °C as a compromise among three factors: inactivation of the fungal strain, dehydration for preservation and analytical purposes, and minimization of oxidation and Maillard reactions. This way, potential negative effects on fungal metabolites are avoided while achieving a dry biomass within 24 h. Additionally, control samples of brown rice (Raw BR) and whole wheat (Raw WW) grains were obtained by milling the grains. These raw flours were used as controls for statistical comparisons and in the figures and discussion, since the objective of this study was to evaluate the potential of fermented flours as a novel ingredient that could replace conventional, non-thermally treated flours in a cookie formulation.

### 2.5. Preparation of Cookies

The cookie dough was prepared following the method described by Blanco Canalis et al. [21], with some modifications. First, 40.50 g of sugar (Chango, BuenosAires, Argentina), 30.30 g of vegetable bakery margarine (Vigras, Buenos Aires, Argentina), 3.39 g of milk (3% fat) powder (La Serenísima, Buenos Aires, Argentina), 0.63 g of salt (Celusal, Buenos Aires, Argentina), and 0.75 g of NaHCO_3_ (Anedra, Buenos Aires, Argentina) were beaten for two minutes at room temperature with an electric mixer (Braun, Kronberg, Germany). Then, water (12.8 g) was added to each formulation, and the mixture was beaten for 1 min. Finally, flour mix (67.8 g) was added and beaten for 2 more minutes at low speed. The resulting dough was manually rolled out to a thickness of 0.7 cm, cut into 4.5 cm diameter circles, and placed on an aluminum baking sheet [23]. The cookies were baked for 11 min at 180 ± 6 °C in a forced convection oven (Pauna, Buenos Aires, Argentina) equipped with a temperature controller. After baking, the cookies were cooled down to room temperature (~20 °C) and wrapped in sealed plastic bags prior to analysis. Two batches per formulation were produced, each yielding nine cookies. From these, the six cookies were randomly selected for quality analysis. Five different formulations were produced: a control cookie made with 100% all-purpose commercial wheat flour (WF) (Gastaldi™) and four variations in which 20% of the wheat flour was replaced with fermented alternatives. The fermented dry biomass ingredients used were *Pleurotus ostreatus*-fermented brown rice flour (POBR), *Ganoderma sessile*-fermented brown rice flour (GSBR), *Pleurotus ostreatus*-fermented whole wheat flour (POWW), and *Ganoderma sessile*-fermented whole wheat flour (GSWW).

### 2.6. Chemical Characterization of Flours and Cookies

#### 2.6.1. Preparation of Extracts

Extracts were prepared following Puttaraju et al. [25] with some modifications. One gram of each sample (flour or ground cookie of each batch) was mixed with 5 mL of distilled water or 5 mL of absolute ethanol. Samples were stirred at room temperature for 30 min in a vortex (XH-D, Faithful Instrument, Tianjin, China) at 1000 rpm to ensure effective extraction, then centrifuged (TDL80-2D, Arcano, Shanghai, China) at 2000× *g* for 15 min at room temperature. Supernatants were stored at 4 °C until completion of the analysis and are referred to as the water extract and the ethanolic extract. Flours with different moisture contents were dried to a constant weight before preparing the extracts.

#### 2.6.2. Total Phenol Content

Phenol content of the water extracts was determined by using the Folin–Ciocalteu colorimetric method [26]. Briefly, 10 µL of extract was mixed with 200 µL of Folin–Ciocalteu reagent and 2.0 mL of distilled water. Subsequently, 800 µL of sodium carbonate solution (15.9%, *w*/*v*) was added. The reaction mixture was incubated at 50 °C for 5 min, allowed to cool to room temperature, and the absorbance was measured at 765 nm in a spectrophotometer (Spectrum SP-2000, Ningbo, China). The results were expressed in mg of gallic acid (mg GA equivalents) per g of sample, using a calibration curve of gallic acid (0.0007–0.005 mg/mL). Analyses were performed in duplicate.

#### 2.6.3. Triterpenoid Content

Triterpenoid content was determined by using the colorimetric method described by Bidegain et al. [27], modified as follows: After transferring 50 μL aliquots of ethanolic extract to 10 mL test tubes, the solvent was evaporated in open tubes in an oven at 60 °C, and 0.5 mL of 70% (*v*/*v*) perchloric acid was added to each tube. The mixtures were heated in a water bath (Vicking, Buenos Aires, Argentine) at 60 °C for 10 min, cooled at room temperature, and diluted with 2.5 mL of acetic acid and 0.15 mL of 5% (*w*/*v*) vanillin (W310700; Sigma, Saint Louis, MO, USA)–acetic acid solution. The absorbance was measured at 548 nm. The total triterpenoid-equivalent signal content was expressed in mg ursolic acid equivalents per g of dry matter using a calibration curve of ursolic acid (0.0006–0.005 mg/mL). Analyses were performed in duplicate.

#### 2.6.4. Main Nutritional Component Assay

Water-soluble extracts from both flours and ground cookies, obtained as described in Section 2.6.1, were used to evaluate the content of soluble protein and reducing sugar. For both assays, 10 µL aliquots of the same water-soluble extract were used. The water-soluble protein content was determined by using the Lowry protein method modified by Peterson [28], while the reducing sugar content was quantified by using the Somogyi–Nelson reaction [29]. Additionally, moisture, ash, lipid, protein (calculated as N × 6.25), and total carbohydrate contents were determined for both flour and ground cookie according to standard methods [30]. Analyses were performed in duplicate. Carbohydrates were determined by difference, calculated as follows: % Carbohydrates = 100 − (ash + fat + total protein). All compositional data are expressed as % (*w*/*w*) on a dry weight basis. Energy value was calculated using the Atwater general factor system, applying conversion factors of 4 kcal/g for protein and carbohydrates and 9 kcal/g for fat [31].

#### 2.6.5. Analysis of Antioxidant Activity In Vitro

(a) Potassium ferricyanide assay

The antioxidant activity was determined using the potassium ferricyanide assay described by Bibi Sadeer et al. [32]. In brief, 0.5 mL of water extract was mixed with 0.5 mL phosphate buffer (0.2 M, pH 6.6) and 0.5 mL potassium ferricyanide (1% *w*/*v*). The resulting mixture was incubated at 50 °C for 20 min. After the incubation period, 0.5 mL trichloroacetic acid (10% *w*/*v*), 2.5 mL deionized water, and 0.5 mL ferric chloride (0.1% *v*/*v*) were added to the mixture. The absorbance of the samples was read at 700 nm. The results were expressed as mg ascorbic acid equivalent per g of dry weight (mg AA/g) using a calibration curve of ascorbic acid (0.002–0.03 mg/mL). Analyses were performed in duplicate.

(b) Scavenging ability according to DPPH radical assay

The scavenging activity against DPPH radicals was determined as follows. An aliquot of 10 µL of ethanolic extract was mixed with 490 µL of absolute ethanol and 1 mL of an ethanolic solution containing DPPH radicals, resulting in a final reaction volume of 1.5 mL and a final concentration of 0.1 mM DPPH in the reaction mixture. The mixture was shaken vigorously and allowed to stand for 30 min in the dark. The absorbance was then measured at 517 nm using a UV–visible spectrophotometer (Spectrum SP-2000 UV, Ningbo, China) [33]. The results were expressed as mg ascorbic acid equivalent per g of dry weight (mg AA/g) using a calibration curve of ascorbic acid (0.0006–0.01 mg/mL). Analyses were performed in duplicate.

#### 2.6.6. Physical Characterization of Flour Samples

The pasting properties of the fermented flour samples were determined using a Rapid Visco Analyzer (RVA series 4500, Perten Instruments, Stockholm, Sweden). Flour samples (3.5 g) were mixed with 25 g of distilled water in an RVA aluminum canister. The dispersions were stirred at 960 rpm for 10 s followed by constant stirring at 160 rpm until the end of the assay, with temperature maintained at 50 °C for 1 min, increased to 95 °C at minute 5 and maintained for 2.5 min, cooled to 50 °C at minute 3, and finally held at 50 °C for 2 min. The pasting profiles of the samples were analyzed using the Standard 1 method in Thermocline for Windows TCW 3.17.3.509 software (RVA series 4500, Perten Instruments). Key parameters such as peak viscosity (PV), breakdown (BD), final viscosity (FV), and setback (SB) were determined from the pasting profiles [34]. Measurements were performed in duplicate.

### 2.7. Physical Characterization of the Cookies

(a) Moisture

The moisture of the cookies was determined using the standard method (AACC 44-15.02) [30]. Analyses were performed in duplicate. Their diameters and thickness were measured using a millimeter scale ruler (Pizzini, Buenos Aires, Argentine) after baking and cooling.

(b) Cookie factor

The cookie factor (CF) [35] was calculated according to the ratio between the total length and total thickness of four units (the most homogeneous ones) chosen from each batch as follows:

$$CF=\frac{\left(D1+D2\right)}{2A}$$

The cookies were put side by side, and the total length (D1) was the sum of the diameters. They were rotated 90°, and the total length (D2) was measured. The cookies were then stacked, and the total height was determined (A).

(c) Texture

The texture of the cookies was analyzed 24 h after baking using a three-point break test on an INSTRON texturometer (Universal Testing Machine, model 3342, Norwood, MA, USA), following Sciarini et al. [36] with some modifications. The samples were placed on two parallel supports, spaced 0.036 m (d) apart. A bar shifted vertically (speed of 0.5 mm/s), exerting a compressive force until the cookie fractured. This procedure was performed on four cookies of each batch. The following parameters were obtained: maximum breaking force (FM), which represents the hardness of the cookie; and breaking stress (σ), which is the pressure exerted on the cookie to break, calculated according to the following equation:

$$\sigma =\frac{3\times FM\times d}{2D\times h2}$$

where d is the span length (0.036 m), D is the cookie diameter, and h is the cookie thickness.

The color of the flours and cookies was determined according to Hrušková et al. [37] using a spectrophotometer with a D65 illuminant at a 10° observation (Minolta 508d, Ramsey, NJ, USA). About 10 g of each flour was spread in a Petri dish, and measurements were taken at three equidistant points. The results were expressed using CIELAB parameters (L^∗^, a^∗^, and b^∗^). The surface color of the cookies was determined after 24 h of baking with a spectrophotometer (Va CM-600d; Konica Minolta, Tokyo, Japan). Analyses were performed in triplicate.

The surface structure of cookies was evaluated using image analysis with ImageJ 1.54m (National Institute of Health, USA), as described by Blanco Canalis [21].

### 2.8. Statistical Analysis

Data were analyzed using mixed models in InfoStat 2020 [38]. Flour formulation was included as a fixed effect. The experimental unit was the flour sample for flour analyses and the cookie batch for cookie analyses. All determinations were performed in duplicate, and duplicate measurements were entered as independent observations in the model, with analytical replication accounted for within the residual variance. Treatment means were compared using Fisher’s least significant difference (LSD) test, and differences were considered significant at *p* < 0.05.

## 3. Results and Discussion

### 3.1. Fermented Flour Characterization

#### 3.1.1. Nutritional Profile

**Figure 1:**
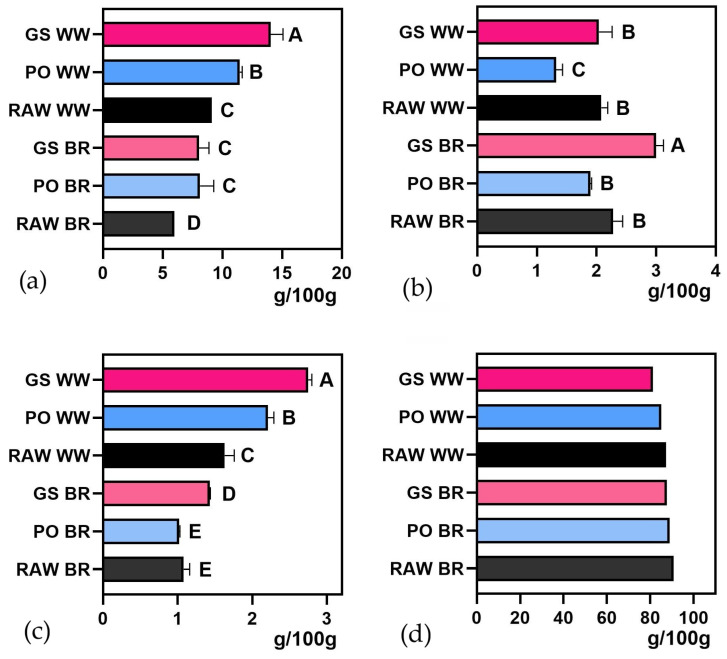
Proximal composition of *Ganoderma sessile*-fermented brown rice (GS BR) and whole wheat (GS WW) and *Pleurotus ostreatus*-fermented brown rice (PO BR) and whole wheat (PO WW) compared with unfermented controls (RAW WW and RAW BR): (**a**) total protein content, (**b**) total fat, (**c**) ash content, and (**d**) carbohydrate content. Different letters indicate significant differences (*p* < 0.05).

**Figure 2:**
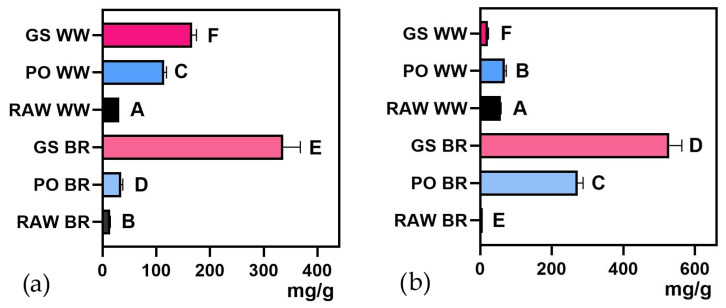
(**a**) Soluble protein content and (**b**) reducing sugar content of *Ganoderma sessile*-fermented brown rice (GS BR) and whole wheat (GS WW) and *Pleurotus ostreatus*-fermented brown rice (PO BR) and whole wheat (PO WW) compared with unfermented controls (RAW WW and RAW BR). Different letters indicate significant differences (*p* < 0.05).

Fermentation enhanced the nutritional profile of grains by increasing their total (Figure 1a, Table S1 of Supplementary Materials) and soluble protein (Figure 2a, Table S1) contents. Total protein increased up to 35% in fermented brown rice and up to 54% in fermented whole wheat, and soluble protein increased up to 2250% and 438% in fermented brown rice (BR) and whole wheat (WW) respectively, compared to raw brown rice and whole wheat flours (RAW BR and RAW WW). Total fat decreased significantly in WW treated with PO, whereas it increased in brown rice treated with GS (Figure 1b, Table S1). Ash content increased significantly in most of the fermented substrates (Figure 1c, Table S1), and carbohydrates decreased slightly (<10%) in all of them (Figure 1d, Table S1). Absolute values of all variables (mean and standard deviation) for all treatments, including those of sterilized non-inoculated grains (S BR, and S WW), are presented in Table S1.

Both mushroom species increased the reducing sugar content of brown rice (Figure 2b), whereas in whole wheat, the reducing sugar content increased with PO but decreased with GS (Figure 2b, Table S1). Reducing sugar determinations according to Lowry’s method can be biased by phenolic or reducing compounds. However, the different trends observed in the polyphenol and reducing sugar contents in some of the flours (e.g., GSWW) suggest that our results are not significantly biased.

The increase in total protein observed in our study varied from 25% to 54%, depending on the substrate and fungal species used. The enhancement in protein content achieved through *P. ostreatus* fermentation is consistent with findings from similar studies. For instance, Asensio-Grau et al. [39] reported an 18.5% increase in protein content in lentils fermented with PO, while He [40] reported a 27.8% increase in protein content in soybeans fermented with PO. Additionally, Eliopoulos [41] observed a 49.49% increase in brewer’s spent grain fermented with PO. Interestingly, *G. sessile* showed significant improvements in the protein content of fermented substrates. Notably, the increase in protein content for whole wheat grains (54%) was higher than most previously reported values for other fungal species. This is especially interesting in the case of *Ganoderma* spp. since most research has concentrated on their bioactive compounds and metabolites of biomedical interest, but the details of their nutritional potential have not yet been explored in depth. It should be highlighted that increases in protein content are based on total nitrogen determination via the Kjeldahl method, without specific assessment of the contribution of non-protein nitrogen, including that associated with structural components of the fungal cell wall.

The increase in protein content in fermented substrates can be attributed to the conversion of plant biomass into fungal biomass and carbon dioxide as a byproduct of respiration. Previous studies have shown that increased total protein content in the substrate is directly associated with mass loss [42] and that mass loss is directly associated with fungal biomass produced [43]. Thus, protein increases are due to both the conversion of substrate into fungal biomass and the concentrating effect resulting from dry mass loss during respiration. This is expected because fungi cannot fix atmospheric nitrogen. Since total protein is measured by total nitrogen content and there is no new nitrogen fixation, any increase in measured protein reflects the concentration of nitrogen as carbon is lost. This explains the remarkable increase in protein after GS fermentation, as this species was shown to cause greater mass loss than PO [12]. Additionally, the considerably higher levels of soluble protein might indicate improved protein bioavailability in fermented substrates, given the positive relationship between protein solubility and digestibility [44,45]. White-rot fungi depolymerize plant cell wall components and assimilate nitrogen compounds from the substrates, thereby altering solubility and enhancing the degradability of plant biomass [46]. Further research, including studies on digestibility and related aspects, is needed to accurately assess the extent of the improvement in the protein bioavailability of fermented substrates.

Our results show that fat content decreased in WW fermented with PO, while it increased in BR treated with GS. These differences may be attributed to the specific metabolic activities of each fungus and its interaction with the nutrients in the substrate. According to Stoffel et al. [6], macronutrient variations during fermentation are influenced by the metabolic capacities of microorganisms and the process conditions. The reduction observed with PO could be explained by its ability to degrade lipids through lipase activity, using them as an energy source during mycelium proliferation [47,48]. In contrast, the increase in GS might be attributed to the synthesis of secondary lipids or their accumulation within the mycelium. Dwivedi et al. [49] reported that some fungi can synthesize fatty acids during cultivation. The decrease in carbohydrates is in line with the findings of similar studies [6,39].

#### 3.1.2. Functional Profile

**Figure 3:**
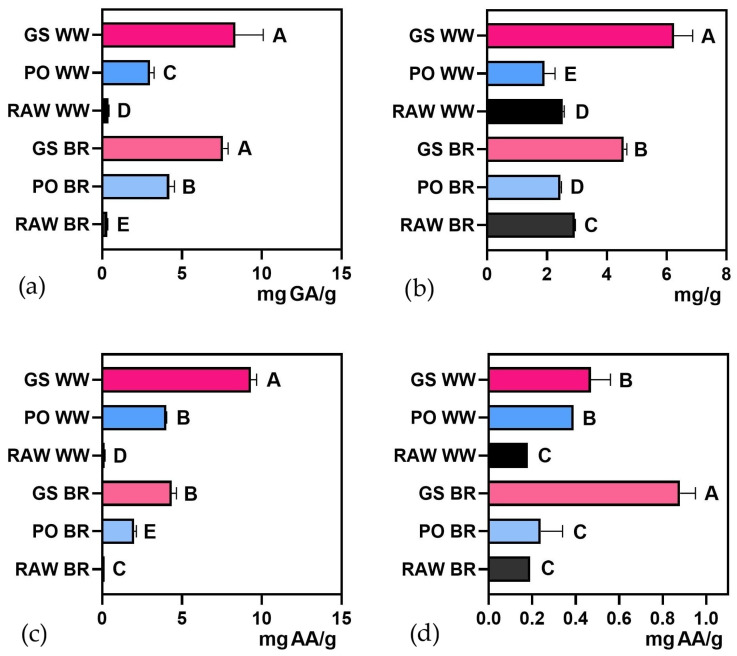
(**a**) Total polyphenol content, (**b**) triterpene content, (**c**) reducing power, and (**d**) DPPH radical scavenging ability of *Ganoderma sessile*-fermented brown rice (GS BR) and whole wheat (GS WW) and *Pleurotus ostreatus*-fermented brown rice (PO BR) and whole wheat (PO WW) compared with unfermented controls (RAW WW and RAW BR). Different letters indicate significant differences (*p* < 0.05).

The total polyphenol content and ferricyanide reducing power of brown rice (BR) and whole wheat (WW) substrates fermented by GS and PO strains exhibited a significant increase compared to raw brown rice and whole wheat flours (RAW BR and RAW WW). (Figure 3a,c; Table S2 of Supplementary Materials). However, the triterpene content increased significantly only in the substrates fermented by the GS strain (Figure 3b; Table S2), but not in PO-fermented substrates. The high triterpene content in GS-fermented substrates aligns with the medicinal properties of this genus, which is known for its bioactive triterpenoids, such as ganoderic and lucidenic acids. The free radical scavenging capacity significantly increased in all fermented substrates compared to the unfermented ones, except for the BR fermented by the PO strain (Figure 3d). Absolute values of all variables (mean and standard deviation) for all treatments, including those of sterilized non-inoculated grains (S BR, and S WW), are presented in Table S2. It should be noted that the Folin–Ciocalteu method can overestimate total polyphenol content since it is not specific to polyphenols and can react with other reducing compounds. Nevertheless, this is the most widely used method in laboratories worldwide to quantify total polyphenol content [50].

Fermented flours showed significantly higher polyphenol content, antioxidant capacity, and free radical scavenging capacity than the unfermented flours. As found in a previous study [12], GS-fermented substrates yielded greater polyphenol levels and antioxidant activity than PO-fermented ones. In addition, GS also led to an increase in triterpenoid content. Similar trends have been reported in other studies on SSF with fungi, emphasizing its potential to boost the antioxidant activity of various substrates [12,51]. This increase could be attributed to the degradation of bound phenolic acids, lignin, and other insoluble polyphenols into smaller phenolic units by laccases and other ligninases [52], which have been shown to enhance antioxidant activities [53,54]. Additionally, hydrolytic enzymes such as α-amylase and β-glucosidase play a crucial role in phenolic mobilization during solid-substrate colonization [55,56]. Previous studies have extensively discussed these mechanisms, reinforcing the enzyme’s role in the observed enrichment of bioactive compounds [57,58,59].

Brown rice and whole wheat are excellent substrates for fermentation, offering an optimal environment for fungal growth [60,61]. Fermented grains consistently show improved nutritional and functional properties compared to non-fermented ones [12,62]. However, the extent of these improvements is influenced not only by the specific microorganism used but also by substrate type, as the same species can exhibit different interactions and metabolic responses depending on the cereal [63,64], as was found in this study.

#### 3.1.3. Physical Characterization

Fermented flour moisture ranged between 3.3 and 4.4%, while raw flour had higher values (9.2–9.4%). The low moisture content of the fermented flours is due to the drying process after fermentation and cannot be attributed to fermentation.

Color analysis results for fermented and unfermented flours dried at 60 °C are presented in Table 1. The darker color observed in the fermented and dried samples can be attributed to chemical changes associated with the lower starch and higher fiber contents due to the fermentation process, as well as to the effects of thermal processes on carbohydrates and proteins during drying. At this temperature, Maillard reactions may occur between reducing sugars and amino acids, leading to the formation of melanoidins, which impart a darker color to the flours [65]. In this context, the increase in reducing sugar content observed in fermented samples (Figure 2) indicates a higher availability of Maillard reaction precursors, which may contribute to the color differences observed after cooking. However, the final color development is likely influenced by the combined effect of sugar availability, protein composition, and structural changes induced by fermentation. Additionally, the drying temperature (~60 °C) of fermented matrices may have promoted some oxidation of phenolic compounds, further contributing to the darker appearance. These thermally induced changes are consistent with observations in other studies in which similar drying temperatures resulted in darker-colored flours due to chemical and structural modifications in the substrate [66].

The Rapid Visco Analyzer (RVA) results showed that fermented flours exhibited starch pregelatinization due to the sterilization treatment conducted before inoculation (Table S3 of Supplementary Materials). This observation suggests structural changes in the starch, as well as chemical reactions induced by the thermal treatment.

### 3.2. Cookie Characterization

**Figure 4:**
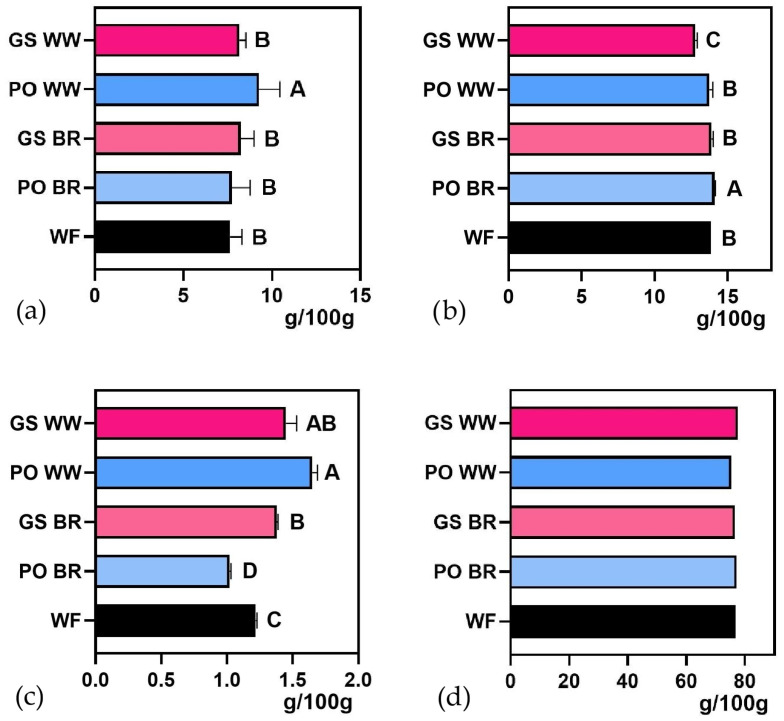
Proximal composition of cookies prepared with 20% *Ganoderma sessile*-fermented brow rice (GS BR), *G. sessile*-fermented whole wheat (GS WW), *Pleurotus ostreatus*-fermented brown rice (PO BR) and *P. ostreatus*-fermented whole wheat (PO WW) compared with the control cookies produced solely with 000 wheat flour (WF): (**a**) total protein content, (**b**) total fat, (**c**) ash content, and (**d**) carbohydrate content. Different letters indicate significant differences (*p* < 0.05).

**Figure 5:**
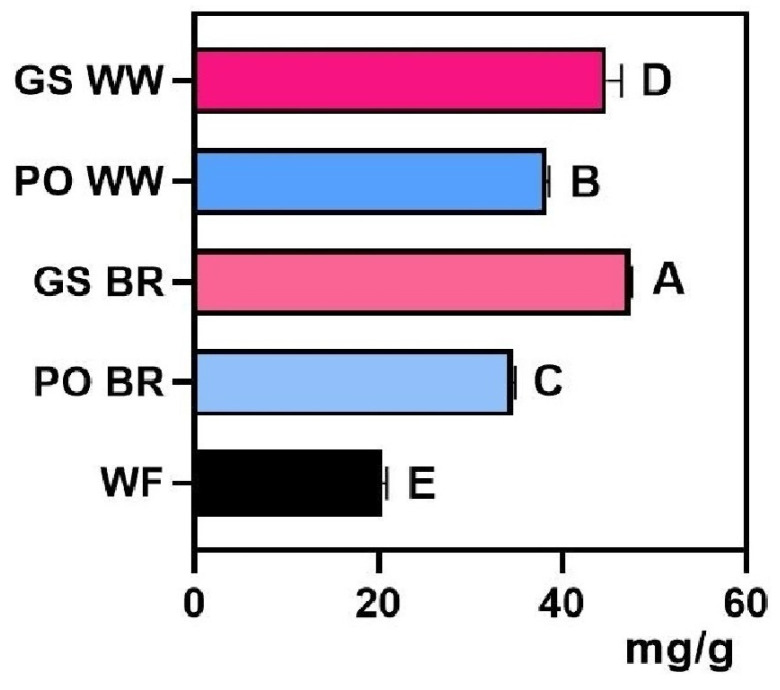
Soluble protein content of cookies prepared with 20% *Ganoderma sessile*-fermented brow rice (GS BR), *G. sessile*-fermented whole-wheat (GS WW), *Pleurotus ostreatus*-fermented brown rice (PO BR), and *P. ostreatus*-fermented whole wheat (PO WW) compared with the control cookies produced solely with 000 wheat flour (WF). Different letters indicate significant differences (*p* < 0.05).

**Figure 6:**
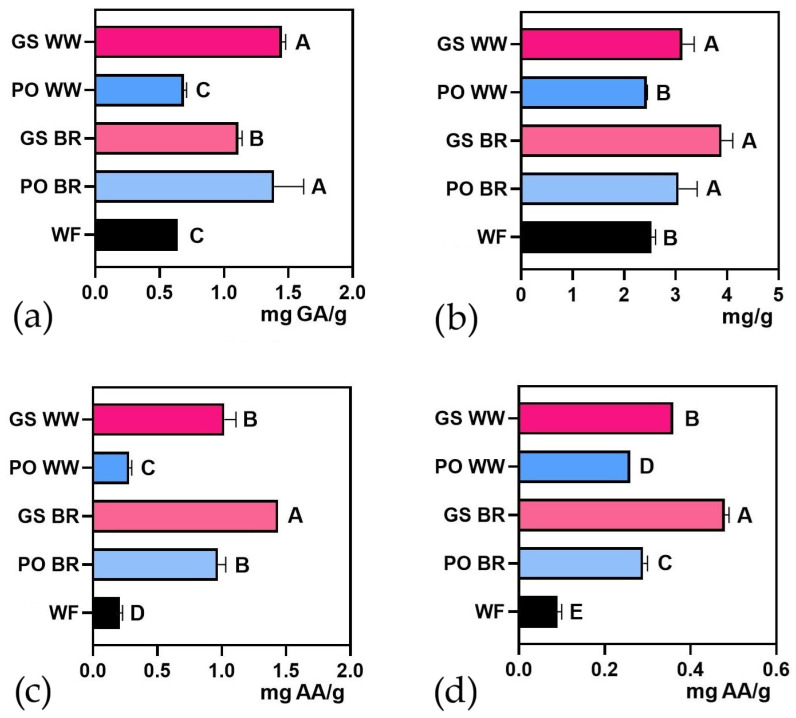
(**a**) Total polyphenol content, (**b**) triterpene content, (**c**) reducing power, and (**d**) DPPH radical scavenging ability of cookies prepared with 20% *Ganoderma sessile*-fermented brow rice (GS BR), *G. sessile*-fermented whole wheat (GS WW), *Pleurotus ostreatus*-fermented brown rice (PO BR), and *P. ostreatus*-fermented whole wheat (PO WW) compared with the control cookies produced solely with 000 wheat flour (WF). Different letters indicate significant differences (*p* < 0.05).

The proximate composition and functional properties of cookies made with fermented and unfermented grains are summarized in Figure 4, Figure 5 and Figure 6 and Table S4 of the Supplementary Materials. The total protein content of cookies (Figure 4a) ranged from 7.6% (*w*/*w*) for the control cookies produced solely with 000 wheat flour (WF) to 9.28% (*w*/*w*) for cookies made with 20% PO-fermented WW. The addition of fermented flours led to a slight increase in the total protein of the cookies; however, only samples containing PO WW showed a significant increase (approximately 21%), compared to those made with WF. Ash content (Figure 4c) generally increased in most cookies produced with fermented grains, except for those prepared with PO BR. Meanwhile, total fat (Figure 4b) and carbohydrate contents (Figure 4d) remained largely stable, primarily due to the influence of the base cookie formulation. Total fat ranged between 12.78% and 14.10% driven by the vegetable fat added to the formulation, and total carbohydrate content ranged between 75.3% and 77.6%. Absolute values of all variables (mean and standard deviation) are presented in Table S4.

Soluble protein content significantly increased in all cookies formulated with fermented flours compared to those prepared with wheat flour, as shown in Figure 5 and Table S4. The increase in soluble protein observed in the fermented flours was also reflected in the cookies produced with these ingredients, indicating that fermentation-induced changes in protein solubility are not completely lost during baking. Although thermal processing can induce protein denaturation, controlled heat treatments such as baking may promote structural rearrangements that preserve protein functionality [67]. This protein reorganization could explain why soluble protein fractions persist in cereal-based baked products. This behavior is consistent with previous studies on biscuits made from fermented flours, in which increases in soluble protein detected at the flour level were retained after the baking process [68].

From a functional perspective, the total polyphenol and triterpene contents (Figure 6a,b and Table S5) significantly increased in cookies containing fermented flours, except for those with PO WW. The reducing power and antioxidant capacity (Figure 6c,d) increased significantly in all cookies containing fermented flours. Absolute values of all variables (mean and standard deviation) are presented in Table S5.

Table 2 shows various cookie quality parameters. The moisture content of sugar-snap cookies is crucial for determining their texture and shelf life. Cookie moisture ranged between 4.1 and 5.4%, which is expected for this kind of product, and did not show significant differences among treatments. This suggests that the initial variations in the moisture content of the flours did not affect this parameter in the final product.

Cookie factor (CF), defined as the width-to-thickness ratio, influences how dough spreads during baking, which in turn determines the cookie’s final shape and texture. The CF is determined by a complex interplay of ingredient ratios and baking conditions; specifically, the type and balance of fat and sugar affect the spread, with higher amounts promoting wider, thinner cookies, while flour and ingredients that retain water provide structure [24]. The cookie factor of the cookies ranged from 5.1 to 6.2 (mm/mm). Cookies made with PO-fermented flour obtained the highest values; however, no significant differences (*p* > 0.05) were observed among cookies from the different treatments.

In terms of appearance, cookies made with fermented flours displayed darker and redder colors, as indicated by the lower and higher values of L* and a* coordinates (Table 2).

The three-point break test was conducted to analyze the force required to bend and break the cookie. Cookie hardness is a result of the intricate interplay between several factors, including the structure, the distribution of fat, starch, and protein, and the moisture content. Additionally, the hardness of the cookie depends on the strength and dimensions of the samples used in the test. The bending test measures the force needed to bend and snap brittle foods such as cookies and crackers. Maximum breaking force (FM) and breaking stress (*σ*) ranged between 55 and 71 N and 510 and 618 kPa, respectively. The highest values of both parameters were obtained by flour fermented with GS WW and WF; however, no significant differences (*p* > 0.05) were found among samples.

Our results indicate that incorporating filamentous fungi-fermented flours into cookie recipes generally enhances their nutritional and functional attributes. However, these improvements do not always align with the patterns observed in the original fermented flours. This discrepancy may be due to the proportion of these ingredients used in the formulation (viz. 20%) and the effects of the baking process. For example, while the antioxidant properties of the cookies were significantly enhanced, the specific patterns of polyphenols and other bioactive compounds did not fully reflect those present in the fermented flours. Factors such as sucrose and glucose content, baking time, and temperature—along with potential thermal degradation, polymerization, oxidation, and interactions within the food matrix—played a notable role in modulating the final presence and activity of these compounds [69,70,71]. These findings highlight the critical influence of baking conditions on the nutritional and functional profile of the final products.

Regarding the physical quality parameters, both cookie factor and hardness remained within expected ranges and showed no significant differences across treatments. This suggests that incorporating 20% fermented flours did not substantially affect these characteristics. However, cookies made with fermented flours exhibited a darker and redder color, likely due to the natural color of the fermented flours and the increased Maillard and caramelization reactions during baking [72].

The pregelatinization and hydrolysis of starch in the fermented flours, as evidenced by the drastic reduction in RVA pasting parameters (Table S3), fundamentally altered their water-binding and viscosity-forming capacity. In sugar-snap cookie systems, where gluten development is deliberately minimized and the structure is primarily supported by a continuous phase of sugar and fat, the role of starch is less dominant in defining final hardness and spread [59]. The cookie matrix is a composite in which sugar syrup and fat form the binding continuum upon cooling, while starch and protein particles act as fillers. The modified, low-viscosity starch from fermented flours likely integrated into this matrix as inert filler particles without disrupting its overall mechanical integrity, given the substitution level was not high enough to critically alter the dough’s rheology.

The lack of significant textural differences is a validation of the technological feasibility of the 20% substitution. It demonstrates that the profound biochemical modifications induced by fungal fermentation—while successfully enhancing nutritional and functional profiles—can be incorporated without compromising the key physical attributes expected of a commercial sugar-snap cookie. This balance is crucial for consumer acceptance. Future studies exploring higher substitution levels or different product matrices would likely reveal more pronounced textural impacts of these modified flours, as the structural role of starch would become more critical.

## 4. Conclusions

This study demonstrates that fermenting whole wheat and brown rice with filamentous fungi, specifically *P. ostreatus* and *G. sessile*, significantly enhances their nutritional and functional properties. These improvements include a substantial increase in total and soluble protein content, suggesting improved protein bioavailability. The results presented here highlight the potential of fungal fermentation as an effective strategy for enriching the nutritional and functional profiles of cereal flours. However, the specific outcomes are modulated by both the fungal species and the substrate type.

Incorporating flours fermented with filamentous fungi presents a promising opportunity for developing nutritionally enhanced cookies with improved functional properties. While the benefits are clear, this study highlights the complex interplay among fermentation, flour incorporation levels, and baking conditions in shaping the final product’s characteristics. Further research, particularly on the bioavailability and digestion of these enhanced compounds, along with formulation and baking process optimization, is essential to fully harness the potential of fermented flours to maximize nutritional and functional benefits in food products.

## Figures and Tables

**Table 1:** Color properties of fermented vs. non-fermented substrates.

Samples	L*	a*	b*
Raw BR	83.42 ± 1.34 ^e^	1.13 ± 0.11 ^a^	14.79 ± 0.30 ^a^
PO BR	67.61 ± 0.41 ^c^	7.18 ± 0.33 ^c^	25.95 ± 0.58 ^c^
GS BR	61.44 ± 0.74 ^b^	9.00 ± 0.36 ^d^	30.68 ± 1.03 ^e^
Raw WW	73.51 ± 2.42 ^d^	4.23 ± 0.33 ^b^	17.49 ± 1.34 ^b^
PO WW	63.18 ± 0.49 ^b^	8.92 ± 0.19 ^d^	28.32 ± 0.58 ^d^
GS WW	59.66 ± 0.16 ^a^	9.00 ± 0.15 ^d^	26.93 ± 0.50 ^c^

Note: Means ± standard deviation followed by different letters are significantly different at *p* < 0.05.

**Table 2:** Properties of cookies produced with fermented vs. non-fermented substrates.

Cookies	L*	a*	b*	Moisture (%)	CF (mm/mm)	FM (N)	σ (kPa)
WF	67.94 ± 3.17 ^c^	5.47 ± 0.57 ^a^	25.66 ± 1.06 ^b^	4.9 ± 0.7 ^a^	5.07 ± 0.47 ^a^	71 ± 8 ^a^	611 ± 110 ^a^
PO BR	51.91 ± 1.91 ^b^	11.35 ± 0.74 ^c^	26.4 ± 1.06 ^c^	4.1 ± 0.5 ^a^	6.05 ± 0.31 ^a^	55 ± 4 ^a^	510 ± 10 ^a^
GS BR	48.84 ± 2.62 ^a^	11.55 ± 0.69 ^c^	26.58 ± 0.65 ^c^	4.9 ± 1.5 ^a^	5.07 ± 0.55 ^a^	64 ± 4 ^a^	540 ± 8 ^a^
PO WW	52.99 ± 2.00 ^b^	11.18 ± 0.65 ^c^	27.21 ± 0.86 ^c^	5.4 ± 1.1 ^a^	6.15 ± 0.42 ^a^	58 ± 11 ^a^	536 ± 85 ^a^
GS WW	47.87 ± 3.39 ^a^	10.51 ± 0.56 ^b^	24.38 ± 0.90 ^a^	5.3 ± 0.1 ^a^	5.90 ± 0.05 ^a^	64 ± 11 ^a^	618 ± 103 ^a^

Note: Means ± standard deviation followed by different letters are significantly different at *p* < 0.05. CF: cookie factor. FM: maximum breaking force. σ: breaking stress.

## Data Availability

All of the original data are included in the article or in the Supplementary Materials; further inquiries can be directed to the corresponding author.

## Supplementary Materials

The following supporting information can be downloaded at https://www.mdpi.com/article/10.3390/foods15030510/s1, Table S1: Nutritional constituents (dry matter) of fermented substrates compared to non-fermented substrates; Table S2: Antioxidant profile and functional components of fermented substrates compared to non-fermented substrates; Table S3: Pasting parameters of fermented substrates compared to non-fermented substrates; Table S4: Nutritional constituents (dry matter) of cookies produced with fermented and unfermented (whole white flour) substrates; Table S5: Antioxidant profile and functional components of cookies with fermented substrates compared to cookies with raw WW. Figure S1: photograph of cookies prepared with the different formulation assayed.

## Abbreviations

The following abbreviations are used in this manuscript:

BR	Brown rice
GS	*Ganoderma sessile*
GS BR	Brown rice fermented by *Ganoderma sessile*
GS WW	Whole wheat fermented by *Ganoderma sessile*
MEA	Malt extract agar culture media
PO	*Pleurotus ostreatus*
PO BR	Brown rice fermented by *Pleurotus ostreatus*
PO WW	Whole wheat fermented by *Pleurotus ostreatus*
S BR	Sterilized non-inoculated brown rice
S WW	Sterilized non-inoculated whole wheat
WF	All-purpose commercial wheat flour
