# Supplementary material for: Nutritional Improvement of Sugar-Snap Cookies Supplemented with Ganoderma sessile and Pleurotus ostreatus Solid-State Fermentation Flours
Source: Foods. 2026 Feb 2;15(3):510. doi: 10.3390/foods15030510 (PMC12896895; doi:10.3390/foods15030510)
Supplement: Supplementary file 1 [file foods-15-00510-s001.zip › foods-4087890-supplementary.pdf]

Supplementary material

Table S1
Nutritional constituents (dry matter) of fermented substrates compared to non-fermented substrates.

Samples	Crude protein (g/100g)	Total fat (g/100g)	Ash (g/100g)	CH (g/100g)	Soluble protein (mg/g)	Reducing sugar (mg/g)
GS WW	14.04±1.04[a]	2.04±0.22[b]	2.75±0.05[a]	81.2	166.48±8.21[f]	21.03±2.61[e]
PO WW	11.46±0.19[b]	1.32±0.11[c]	2.21±0.08[b]	85.0	114.53±4.47[c]	68.98±3.8[c]
Raw WW	9.10±0.00[c]	2.08±0.11[b]	1.63±0.13[c]	87.2	30.90±0.00[a]	58.21±0.11[d]
S WW	9.26 0.28	1.84±0.04	1.68±0.03	87.22	11.69±6.22	3.57±0.17
GS BR	8.04±0.84[c]	3.00±0.12[a]	1.43±0.01[d]	87.6	335.97±32.11[e]	528.11±34.61[a]
PO BR	8.12±1.16[c]	1.90±0.02[b]	1.02±0.01[e]	88.9	34.77±2.78[d]	272.71±14.44[b]
Raw BR	5.99±0.00[d]	2.28±0.16[b]	1.08±0.08[e]	90.7	14.27±0.06[b]	7.34±0.13[f]
S BR	6.89±1.08	7.71±6.44	1.21±0.30	84.19	10.45±0.86	1.39±0.22

Note: Means ± Standard Deviation followed by different letters are significantly different at $p < 0.05$. CH: carbohydrates

GSWW: whole wheat fermented by *Ganoderma sessile* flour
POWW: whole wheat fermented by *Pleurotus ostreatus* flour
RawWW: whole wheat flour
S WW: sterilized, non-inoculated whole wheat
GSBR: brow rice fermented by *Ganoderma sessile* flour
POBR: brow rice fermented by *Pleurotus ostreatus* flour
RawBR: brown rice flour
S BR: sterilized, non-inoculated brown rice

Table S2.
Antioxidant profile and functional components of fermented substrates compared to non-fermented substrates.

Samples	Total phenol (mg GA/g)	Triterpenes (mg/g)	FRP (mg AA/g)	FRSC (mg AA/g)
GS WW	8.34±1.76[a]	6.25±0.62[a]	9.32±0.36[a]	0.47±0.09
PO WW	3.00±0.26[c]	1.92±0.35[e]	4.02±0.03[b]	0.39±0.00[b]
Raw WW	0.40±0.03[d]	2.53±0.05[d]	0.17±0.01[d]	0.18±0.00c[c]
S WW	0.39±0.03	1.11±0.09	0.20±0.02	0.12±0.01
GS BR	7.58±0.31[a]	4.57±0.01[b]	4.36±0.30[b]	0.88±0.07[a]
PO BR	4.20±0.33[b]	2.46±0.02[d]	2.00±0.15[e]	0.24±0.10[c]
Raw BR	0.33±0.02[e]	2.93±0.02[c]	0.16±0.00[c]	0.19±0.00[c]
S BR	0.40±0.14	2.35±0.95	0.24±0.06	0.17±0.01

Note: Means ± Standard Deviation followed by different letters are significantly different at $p < 0.05$.
FRP: Ferricyanide reducing power. FRSC: Free radical scavenging capacity power

GSWW: whole wheat fermented by *Ganoderma sessile* flour
POWW: whole wheat fermented by *Pleurotus ostreatus* flour
RawWW: whole wheat flour
S WW: sterilized, non-inoculated whole wheat
GSBR: brow rice fermented by *Ganoderma sessile* flour
POBR: brow rice fermented by *Pleurotus ostreatus* flour
RawBR: brown rice flour
S BR: sterilized, non-inoculated brown rice

Table S3.
Pasting parameters of fermented substrates compared to non-fermented substrates.

Samples	Pv (cP)	BD (cP)	FV (cP)	SB (cP)	Peak Time (min)	Pasting Temperature (°C)
PO WW	39±0	4±0	69±0	34±0	-	-
GS WW	14±0	2±0	20±1	8±1	-	-
Raw WW	853±0	751±0	1781±0	1030±0	5.4±0	88.85
S WW	115	102	243	141	-	-
GS BR	22±0	4±0	22±0	4±0	-	-
PO BR	18±0	3±0	20±1	5±1	-	-
Raw BR	2809±0	72±0	6597±0	3860±0	7.0±0	84.8
S BR	47±1	42±0.5	71±3	29±2.5	-	-

Note: Pv: peak viscosity, BD: breakdown, FV: final viscosity, SB: setback

GSWW: whole wheat fermented by *Ganoderma sessile* flour
POWW: whole wheat fermented by *Pleurotus ostreatus* flour
RawWW: whole wheat flour
S WW: sterilized, non-inoculated whole wheat
GSBR: brow rice fermented by *Ganoderma sessile* flour
POBR: brow rice fermented by *Pleurotus ostreatus* flour
RawBR: brown rice flour
S BR: sterilized, non-inoculated brown rice

Table S4.
Nutritional constituents (dry matter) of cookies produced with 20% substitution of wheat flour with fermented flours vs cookies made solely with all-purpose commercial wheat flour

Cookies	Crude protein (g/100g)	Total fat (g/100g)	Ash (g/100g)	CH (g/100g)	Soluble protein (mg/g)	Energy value (kcal/100g)
GSWW	8.16±0.37^{b}	12.78±0.14^{c}	1.45±0.08^{ab}	77.61	44.8±1.6^{d}	458.1
PO WW	9.28±1.16^{a}	13.75±0.24^{b}	1.65±0.04^{a}	75.33	38.3±0.3^{b}	462.19
GS BR	8.23±0.76^{b}	13.89±0.13^{b}	1.38±0.01^{b}	76.51	47.4±0.1^{a}	463.97
PO BR	7.73±1.05^{b}	14.10±0.06^{a}	1.02±0.01^{d}	77.15	34.6±0.3^{c}	466.42
WF	7.62±0.65^{b}	13.85±0.1^{b}	1.22±0.01^{c}	76.74	20.4±0.5^{e}	462.09

Note: Means ± Standard Deviation followed by different letters are significantly different at $p < 0.05$. CH: carbohydrates.

GSWW: 20% substitution with whole wheat fermented by *Ganoderma sessile* flour
POWW: 20% substitution with whole wheat fermented by *Pleurotus ostreatus* flour
GSBR: 20% substitution with brow rice fermented by *Ganoderma sessile* flour
POBR: 20% substitution with brow rice fermented by *Pleurotus ostreatus* flour
WF: solely all-purpose commercial wheat flour

Table S5.
Antioxidant profile and functional components of cookies with fermented substrates compared to cookies with raw WW.

Cookies	Total phenol (mgGA/g)	Triterpenes (mg/g)	FRP (mgAA/g)	FRSC (mgAA/g)
GSWW	1.45±0.03[a]	3.13±0.23[a]	1.02±0.09[b]	0.36±0.00[b]
PO WW	0.69±0.02[c]	2.44±0.01[b]	0.28±0.02[c]	0.26±0.00[d]
GSBR	1.11±0.03[b]	3.89±0.22[a]	1.44±0.00[a]	0.48±0.01[a]
PO BR	1.39±0.23[a]	3.06±0.36[a]	0.97±0.06[b]	0.29±0.01[c]
WF	0.64±0.00[c]	2.53±0.08[b]	0.21±0.02[d]	0.09±0.01[e]

Note: Means ± Standard Deviation followed by different letters are significantly different at $p < 0.05$. FRP: Ferricyanide reducing power. FRSC: Free radical scavenging capacity power

GSWW: 20% substitution with whole wheat fermented by *Ganoderma sessile* flour
POWW: 20% substitution with whole wheat fermented by *Pleurotus ostreatus* flour
GSBR: 20% substitution with brow rice fermented by *Ganoderma sessile* flour
POBR: 20% substitution with brow rice fermented by *Pleurotus ostreatus* flour
WF: solely all-purpose commercial wheat flour

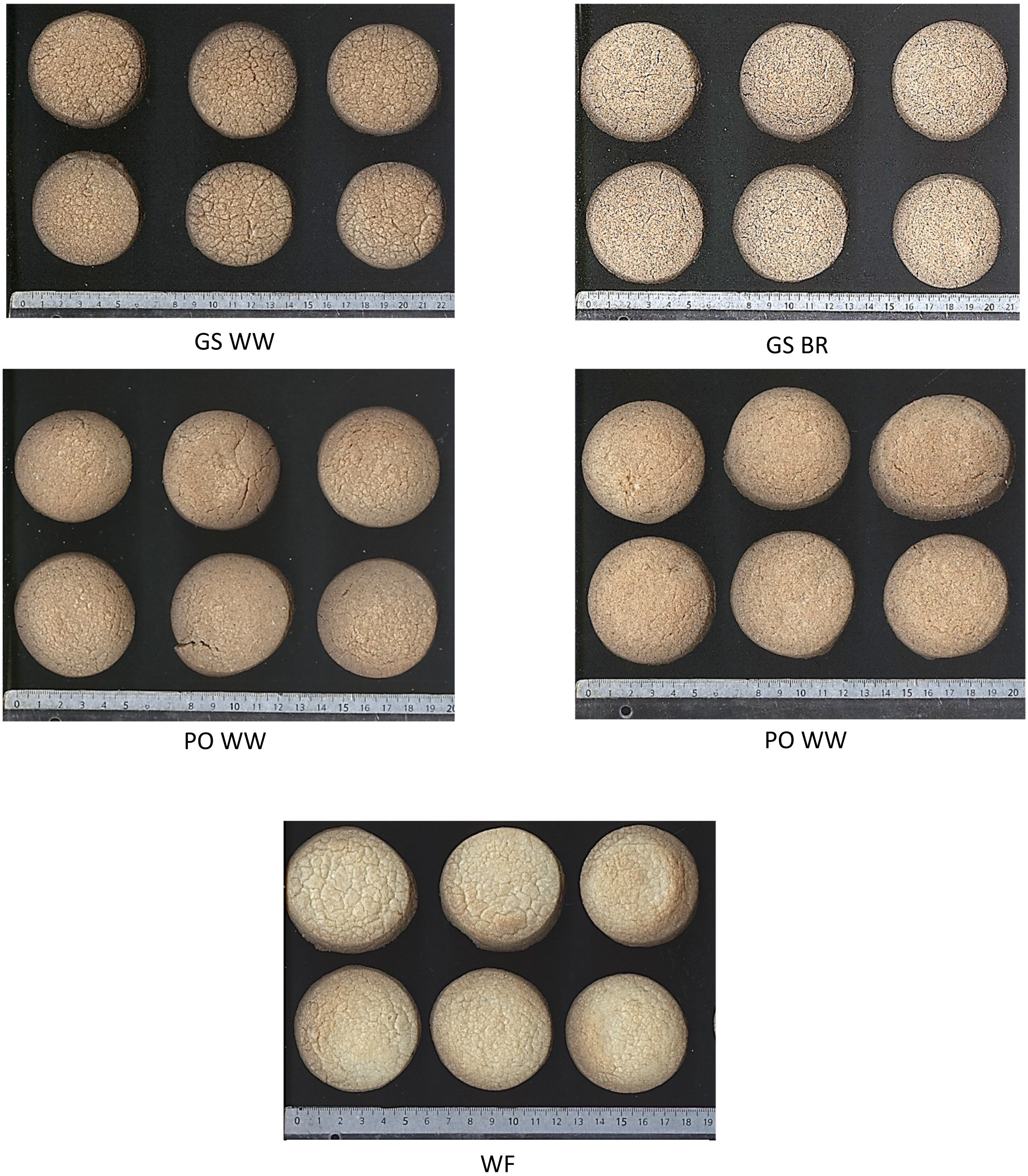

Figure S1: Photographs of the cookies prepared with the different formulations:

GSWW: 20% substitution with whole wheat fermented by *Ganoderma sessile* flour

POWW: 20% substitution with whole wheat fermented by *Pleurotus ostreatus* flour

GSBR: 20% substitution with brow rice fermented by *Ganoderma sessile* flour

POBR: 20% substitution with brow rice fermented by *Pleurotus ostreatus* flour

WF: solely all-purpose commercial wheat flour
